# Comparing the characteristics and predicting the survival of patients with head and neck melanoma versus body melanoma: a population-based study

**DOI:** 10.1186/s12885-021-08105-y

**Published:** 2021-04-16

**Authors:** Yuxin Ding, Runyi Jiang, Yuhong Chen, Jing Jing, Xiaoshuang Yang, Xianjie Wu, Xiaoyang Zhang, Jiali Xu, Piaopiao Xu, Shu Chen LiuHuang, Zhongfa Lu

**Affiliations:** 1 Department of Dermatology, The Second Affiliated Hospital, School of Medicine, Zhejiang University, Hangzhou, China, No. 88, Jiefang Road, Hangzhou, 310009 China; 2 Spinal Tumor Center, Department of Orthopaedic Oncology, Changzheng Hospital, Second Military Medical University, Shanghai, 200003 China; 3 China Academy of Art, Hangzhou, 310000 China

**Keywords:** Cutaneous melanoma, Head and neck melanoma, Nomogram, Prognostic factors, Survival, SEER

## Abstract

**Background:**

Previous studies reported cutaneous melanoma in head and neck (HNM) differed from those in other regions (body melanoma, BM). Individualized tools to predict the survival of patients with HNM or BM remain insufficient. We aimed at comparing the characteristics of HNM and BM, developing and validating nomograms for predicting the survival of patients with HNM or BM.

**Methods:**

The information of patients with HNM or BM from 2004 to 2015 was obtained from the Surveillance, Epidemiology, and End Results (SEER) database. The HNM group and BM group were randomly divided into training and validation cohorts. We used the Kaplan-Meier method and multivariate Cox models to identify independent prognostic factors. Nomograms were developed via the rms and dynnom packages, and were measured by the concordance index (C-index), the area under the curve (AUC) of the receiver operating characteristic (ROC) curve and calibration plots.

**Results:**

Of 70,605 patients acquired, 21% had HNM and 79% had BM. The HNM group contained more older patients, male sex and lentigo maligna melanoma, and more frequently had thicker tumors and metastases than the BM group. The 5-year cancer-specific survival (CSS) and overall survival (OS) rates were 88.1 ± 0.3% and 74.4 ± 0.4% in the HNM group and 92.5 ± 0.1% and 85.8 ± 0.2% in the BM group, respectively. Eight variables (age, sex, histology, thickness, ulceration, stage, metastases, and surgery) were identified to construct nomograms of CSS and OS for patients with HNM or BM. Additionally, four dynamic nomograms were available on web. The internal and external validation of each nomogram showed high C-index values (0.785–0.896) and AUC values (0.81–0.925), and the calibration plots showed great consistency.

**Conclusions:**

The characteristics of HNM and BM are heterogeneous. We constructed and validated four nomograms for predicting the 3-, 5- and 10-year CSS and OS probabilities of patients with HNM or BM. These nomograms can serve as practical clinical tools for survival prediction and individual health management.

**Supplementary Information:**

The online version contains supplementary material available at 10.1186/s12885-021-08105-y.

## Background

Worldwide, cutaneous melanoma is an important public health problem and accounts for 1.7% of all newly diagnosed primary malignant tumors annually [1]. In the United States, the incidence of cutaneous melanoma continues to rise, and the 5-year relative survival rate is 92% (2009–2015) [2]. Many studies have reported that anatomic location is an important prognostic factor for primary cutaneous melanoma [3–5], and head and neck melanoma (HNM) should be treated differently from melanoma in other regions (body melanoma, BM) [6–9]. The head and neck areas are more likely to be exposed to chronic and continued sun exposure, while other body areas usually receive intermittent UV radiation [10, 11]. When considering the cancer density, cutaneous melanoma occurs more frequently in the head and neck regions than in the trunk and extremity regions [7–9]. In addition, some studies have reported poorer survival in HNM patients [12, 13].

To date, limited studies have compared the clinical features of HNM and BM patients based on a large population [9, 14, 15], and few have compared the prognostic factors of the two subsites [16]. Several studies have reported that some clinical features, such as age, sex, histology and thickness, are independent prognostic factors for HNM [14, 17, 18]. However, few studies have developed an integrated tool to comprehensively predict the prognoses of HNM and BM, especially according to different subsites. Therefore, a further understanding of the characteristics and survival of patients with HNM and BM is necessary to aid in clinical management.

A nomogram, which integrates all types of factors to estimate the probability of an event, is a useful graphical predictive tool that can provide the overall probability of a specific outcome for any patient [19, 20]. As a reliable tool used to predict tumor prognosis, the nomogram has been widely used in numerous survival studies [21, 22]. The Surveillance, Epidemiology, and End Results (SEER) database provides information on cancer incidence, treatment and survival from 18 population-based cancer registries, covering 28% of the US population [23]. Many studies have utilized the high-quality database to analyze melanoma characteristics, risks and prognoses [24–26]. Lachiewicz et al. collected SEER data from 1992 to 2003 to examine survival differences between patients with scalp or neck melanoma and those with melanoma of other sites [27]. Yu Xiao et al. used a SEER population to develop a prognostic nomogram for nonmetastatic melanoma patients [28].

In this study, we performed a large SEER population-based study to compare the clinicopathological characteristics and treatments of HNM and BM, aimed at identifying independent prognostic factors of the two subgroups. We attempted to gain a further understanding of the prognosis and develop nomograms as practical clinical tools for predicting the survival of patients with HNM or BM.

## Methods

### Patient selection

The information of eligible patients with malignant cutaneous melanoma from 2004 to 2015 was obtained using SEER*Stat (version 8.3.8) from the SEER database [29]. In general, extracting SEER data does not require informed patient consent, and the SEER database does not provide case-identifying information. As a consequence, approval from the institutional review board is not required.

In this study, the inclusion criteria were as follows: 1) patients had a confirmed diagnosis of malignant cutaneous melanoma with ICD-O-3/WHO 2008 morphology codes 8721–8723, 8726–8728, 8730, 8740–8746, 8760–8761, 8770–8774, 8780 and 8790; 2) the codes of the primary site were C440-C447; 3) patients acquired a diagnosis with a living status; and 4) patients had active follow-up. The exclusion criteria were as follows: 1) clinical diagnosis without pathological reports; 2) acquired diagnosis from autopsy or death certificate only; and 3) unknown survival months. Fifteen variables were extracted from the SEER program, including age, sex, race, histology, anatomic site, thickness, ulceration, stage, metastases, primary site surgery, radiation, chemotherapy, survival time, overall survival (OS) status and cancer-specific survival (CSS) status. We excluded patients who did not have complete information on all the above variables. The stage in the SEER program referred to the stage of lymph nodes, including localized, regional and distant lymph nodes.

CSS was defined as the duration from the first day of follow-up to the day of death due to malignant cutaneous melanoma or the day of the last follow-up, while OS was defined as the time from the first day of follow-up to death from any cause or the end of the follow-up. Patients whose OS was recorded as dead but had 0 survival months in SEER were assigned a survival time of 0.5 months for analysis, while patients alive at follow-up with 0 survival months were excluded. Patients with anatomic site codes C440- Skin of lip, C441- Eyelid, C442- External ear, C443- Skin other/unspecific parts of face, or C444- Skin of scalp and neck were assigned to the HNM group, while those whose site was coded as C445- Skin of trunk, C446- Skin of upper limb and shoulder, or C447- Skin of lower limb and hip were assigned to the BM group.

**Fig. 1:**
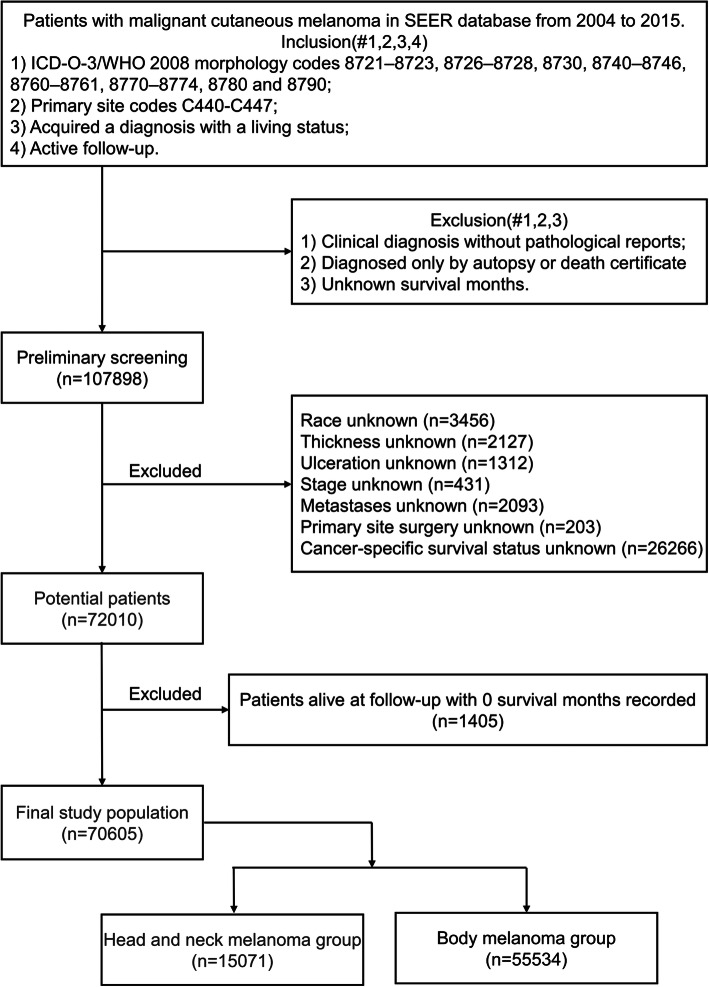
Flowchart of patient selection

Ultimately, a total of 70,605 malignant cutaneous melanoma patients (15,071 HNM patients and 55,534 BM patients) were acquired for further analysis. The patient selection workflow is shown in Fig. 1.

### Statistical analysis

**Fig. 2:**
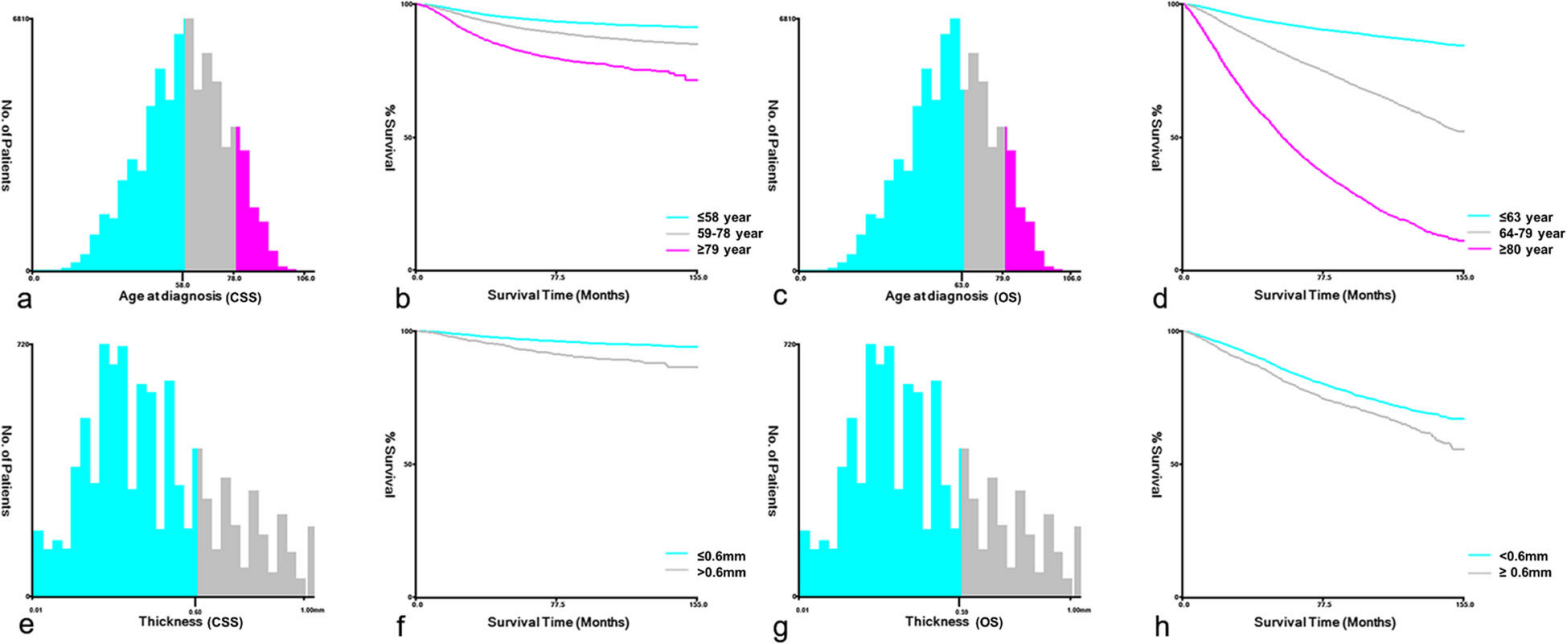
The optimal cut-off values for age (**a**-**b**) based on CSS were ≤ 58, 59–78, and ≥ 79 years old; (**c**-**d**) based on OS were ≤ 63, 64–79, and ≥ 80 years old. The optimal cut-off values for thin HNM (0–1.00 mm) (**e**-**f**) based on CSS were ≤ 0.6 and > 0.6 mm; (**g**-**h**) based on OS were < 0.6 and ≥ 0.6 mm. CSS = cancer-specific survival; OS = overall survival; HNM = head and neck melanoma

Descriptive data were described as frequencies and percentages. X-tile software (Yale University, New Haven, CT, USA) was employed to define the optimal cutoff values of continuous variables [30]. X-tile software divided age into three subgroups for all possible divisions and selected the optimal cutoff value based on the highest chi-square value calculated by Kaplan-Meier survival analysis and the log-rank test. According to CSS, the optimal cutoff values for age were subdivided into ≤58, 59–78 and ≥ 79 years, while based on OS, the optimal cutoff points were ≤ 63, 64–79, and ≥ 80 years (Fig. 2). Melanoma thickness was classified into six subsets (< 0.6 mm, 0.6–0.79 mm, 0.8–1.0 mm, 1.01–2.0 mm, 2.01–4.0 mm, and > 4.0 mm) based on the American Joint Committee on Cancer (AJCC) staging system, the thin melanoma study and X-tile results. The 8th edition of the AJCC staging manual stated an increased risk of fatality in patients with a melanoma thickness of 0.8–1.0 mm [31]. Claeson et al. reported that the risk of fatality in thin melanoma on the scalp was sixfold higher than that on the back [32]. As thin malignant melanomas accounted for a large number of melanomas in our study and scalp location is a risk factor, we explored the cutoff for thin HNM with X-tile, which revealed a thickness of 0.6 mm (Fig. 2).

For nomogram construction and validation, the HNM group and BM group were randomly divided into training and validation cohorts (split ratio: 7:3). The Pearson χ^2^ test or Fisher exact test was performed to compare the baseline characteristics between the HNM group and BM group and to calculate the associations between the training cohort and the validation cohort. *P* < 0.05 was considered significant.

In the univariate analysis, the Kaplan-Meier method and the log-rank test were conducted to determine significant prognostic factors in each of the HNM and BM training cohorts for CSS or OS by SPSS 25.0 (SPSS Inc., Chicago, IL, USA), and factors with *p* < 0.1 were included in the multivariate Cox regression analysis to determine the independent prognostic factors (*p* < 0.05). Furthermore, corresponding hazard ratios (HR) with 95% confidence intervals (CIs) were calculated. For each of the HNM and BM groups, two multivariate Cox models were created to examine the relative effects of variables on CSS or OS. GraphPad Prism 8.0 (GraphPad Software, San Diego, CA, USA) was used to create survival analysis curves for the independent prognostic factors. Notably, as specific information for radiotherapy and chemotherapy was not provided in the SEER database, we did not conduct survival analysis for the two factors.

### Nomogram construction and validation

In the HNM group and BM group, nomograms based on the results of the Cox regression analyses, models were developed for the 3-year, 5-year and 10-year CSS rates via the rms package in R (version 4.0.1). Predictive prognostic nomograms for OS were also constructed for the two groups. In addition, the DynNom package was used to build dynamic nomograms on the web page. The performance and predictive value of the nomograms were measured by the concordance index (C-index) and the area under the curve (AUC) of the receiver operating characteristic (ROC) curve. In addition, calibration plots were used to assess the prediction accuracy of the nomograms by comparing the predicted values with the actual outcomes.

## Results

### Comparison of the characteristics of head and neck melanoma versus body melanoma

A total of 70,605 eligible patients diagnosed with malignant cutaneous melanoma from 2004 to 2015 were evaluated in this study, and the total population was divided into an HNM group and a BM group according to the anatomic site of the tumor.

**Fig. 3:**
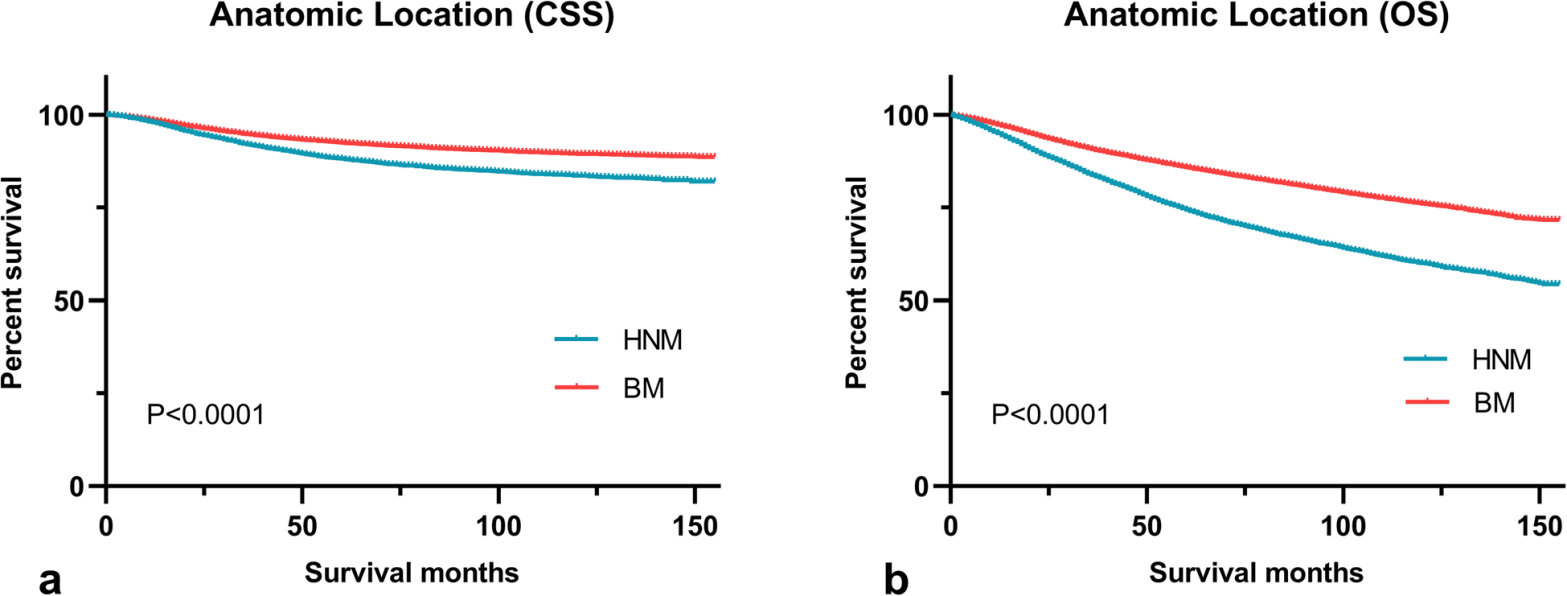
Kaplan-Meier curves of cancer-specific and overall survival based on anatomic location. **a** cancer-specific survival (CSS); **b** overall survival (OS). HNM = head and neck melanoma; BM = body melanoma

The comparison of the two groups is summarized in Table 1. The HNM cohort contained 15,071 (21%) patients, and the BM cohort contained 55,534 (79%) patients. Compared with the BM group, the HNM group contained more older patients (mean age 64.6 vs. 57.2 years) and male patients (72.4% vs. 52.3%). The BM group also comprised a lower proportion of white patients than the HNM group. Superficial spreading melanoma (SSM) was the most common histological type in both groups, but lentigo maligna melanoma (LMM) was much more frequent in the HNM group than in the BM group (31.8% vs. 6.6%). HNM patients were diagnosed with a thicker tumor, more frequently had metastases and were more often treated with radiation than BM patients. The mean survival time in the BM group was longer than that in the HNM group. Additionally, 27.4% of patients with HNM died, whereas 16.2% of patients with BM died. The 5-year CSS and OS rates of HNM patients were significantly lower than those of BM patients. In addition, the Kaplan-Meier curves showed that the prognosis of patients with BM was better than that of patients with HNM (Fig. 3).

**Table 1:** Comparison of head and neck melanoma (HNM) with body melanoma (BM)

	HNM	BM	***P*** value
**Total**	15,071 (21)^c^	55,534 (79)^d^	
**Age, mean (y)**	64.6	57.2	
**Age, median (y)**	67	58	
**Age (n %)**			**< 0.001**
≤ 58	4816 (32.0)	28,941 (52.1)	
59–78	6908 (45.8)	21,114 (38.0)	
≥ 79	3347 (22.2)	5479 (9.9)	
**Sex (n %)**			**< 0.001**
Male	10,913 (72.4)	29,066 (52.3)	
Female	4158 (27.6)	26,468 (47.7)	
**Race (n %)**			**< 0.001**
White	14,928 (99.1)	54,684 (98.5)	
^a^Others	143 (0.9)	850 (1.5)	
**Histology (n %)**			**< 0.001**
NM	2352 (15.6)	7905 (14.2)	
LMM	4798 (31.8)	3638 (6.6)	
SMM	6168 (40.9)	38,748 (69.8)	
^b^Other Melanoma	1753 (11.6)	5243 (9.4)	
**Thickness (n %)**			**< 0.001**
< 0.6 mm	6717 (44.6)	25,301 (45.6)	
0.6–0.79 mm	1453 (9.6)	6772 (12.2)	
0.8–1.0 mm	1148 (7.6)	5226 (9.4)	
1.01–2.0 mm	2394 (15.9)	8746 (15.7)	
2.01–4.0 mm	1759 (11.7)	5337 (9.6)	
> 4.0 mm	1600 (10.6)	4152 (7.5)	
**Ulceration (n %)**			**0.004**
No	12,651 (83.9)	47,153 (84.9)	
Yes	2420 (16.1)	8381 (15.1)	
**Stage (n %)**			**< 0.001**
Localized	12,944 (85.9)	49,320 (88.8)	
Regional	1868 (12.4)	5408 (9.7)	
Distant	259 (1.7)	806 (1.5)	
**Metastases (n %)**			**< 0.001**
No	14,861 (98.6)	54,980 (99.0)	
Yes	210 (1.4)	554 (1.0)	
**Surgery (n %)**			**0.021**
No	342 (2.3)	1092 (2.0)	
Yes	14,729 (97.7)	54,442 (98.0)	
**Radiation (n %)**			**< 0.001**
No/Unknown	14,519 (96.3)	55,055 (99.1)	
Yes	552 (3.7)	479 (0.9)	
**Chemotherapy (n %)**			0.474
No/Unknown	14,869 (98.7)	54,833 (98.7)	
Yes	202 (1.3)	701 (1.3)	
**Survival months, mean**	60.59	67.76	**< 0.001**
**OS (n %)**			**< 0.001**
Alive	10,942 (72.6)	46,547 (83.8)	
Dead	4129 (27.4)	8987 (16.2)	
**CSS (n %)**			**< 0.001**
Alive	13,431 (89.1)	51,448 (92.6)	
Dead	1640 (10.9)	4086 (7.4)	
**5-year OS rate** **(%, mean ± SD)**	74.4 ± 0.4	85.8 ± 0.2	**< 0.001**
**5-year CSS rate** **(%, mean ± SD)**	88.1 ± 0.3	92.5 ± 0.1	**< 0.001**

*NM* nodular melanoma, *LMM* Lentigo maligna melanoma, *SSM* superficial spreading melanoma, *CSS* cancer-specific survival, *OS* overall survival, *SD* standard deviation

*P* values were obtained using the Pearson χ^2^ test or Fisher exact test. Bold values indicate statistical significance less than 0.05

^a^ Others = black, Asian or pacific islander, American indian/Alaska native

^b^ Other melanoma: International Classification of Diseases for Oncology-O-3, codes 8722–8723, 8730, 8740–8741, 8744–8746, 8761, 8770–8774, 8780

^c^HNM constitute 21% of all cutaneous melanomas

^d^BM constitute 79% of all cutaneous melanomas

All HNM patients were randomly divided into training (*n* = 10,551) and validation (*n* = 4520) cohorts. Using the same method, 38,876 BM patients were assigned to the training cohort, and 16,658 BM patients were assigned to the validation cohort. Table 2 shows the characteristics of patients in the training and validation cohorts in each of the HNM and BM groups.

**Table 2:** The characteristics of patients between the training cohort and the validation cohort

	the HNM group			the BM group		
	Training cohort (%)	Validation cohort (%)	***P*** value	Training cohort (%)	Validation cohort (%)	***P*** value
**Total**	10,551 (70.0)	4520 (30.0)		38,876 (70.0)	16,658 (30.0)	
**Age**			0.21			0.748
≤ 58	3349 (31.7)	1467 (32.5)		20,275 (52.2)	8666 (52.0)	
59–78	4885 (46.3)	2023 (44.8)		14,790 (38.0)	6324 (38.0)	
≥ 79	2317 (22.0)	1030 (22.8)		3811 (9.8)	1668 (10.0)	
**Sex**			0.62			0.836
Male	7653 (72.5)	3260 (72.1)		20,359 (52.4)	8707 (52.3)	
Female	2898 (27.5)	1260 (27.9)		18,517 (47.6)	7951 (47.7)	
**Race**			0.911			0.31
White	10,452 (99.1)	4476 (99.0)		38,267 (98.4)	16,417 (98.6)	
^a^Others	99 (0.9)	44 (1.0)		609 (1.6)	241 (1.4)	
**Histology**			0.31			0.067
NM	1680 (15.9)	672 (14.9)		5448 (14.0)	2457 (14.7)	
LMM	3328 (31.5)	1470 (32.5)		2589 (6.7)	1049 (6.3)	
SMM	4306 (40.8)	1862 (41.2)		27,177 (69.9)	11,571 (69.5)	
^b^Other Melanoma	1237 (11.7)	516 (11.4)		3662 (9.4)	1581 (9.5)	
**Thickness**			0.096			**0.012**
< 0.6 mm	4691 (44.5)	2026 (44.8)		17,866 (46.0)	7435 (44.6)	
0.6–0.79 mm	987 (9.4)	466 (10.3)		4711 (12.1)	2061 (12.4)	
0.8–1.0 mm	827 (7.8)	321 (7.1)		3699 (9.5)	1527 (9.2)	
1.01–2.0 mm	1666 (15.8)	728 (16.1)		6073 (15.6)	2673 (16.0)	
2.01–4.0 mm	1268 (12.0)	491 (10.9)		3666 (9.4)	1671 (10.0)	
> 4.0 mm	1112 (10.5)	488 (10.8)		2861 (7.4)	1291 (7.8)	
**Ulceration**			0.459			0.43
No	8841 (83.8)	3810 (84.3)		33,040 (85.0)	14,113 (84.7)	
Yes	1710 (16.2)	710 (15.7)		5836 (15.0)	2545 (15.3)	
**Stage**			0.795			0.159
Localized	9051 (85.8)	3893 (86.1)		34,591 (89.0)	14,729 (88.4)	
Regional	1320 (12.5)	548 (12.1)		3731 (9.6)	1677 (10.1)	
Distant	180 (1.7)	79 (1.7)		554 (1.4)	252 (1.5)	
**Metastases**			0.937			0.251
No	10,405 (98.6)	4456 (98.6)		38,501 (99.0)	16,479 (98.9)	
Yes	146 (1.4)	64 (1.4)		375 (1.0)	179 (1.1)	
**Surgery**			0.545			0.591
No	245 (2.3)	97 (2.1)		773 (2.0)	319 (1.9)	
Yes	10,306 (97.7)	4423 (97.9)		38,103 (98.0)	16,339 (98.1)	

*HNM* head and neck melanoma, *BM* body melanoma, *NM* nodular melanoma, *LMM* Lentigo maligna melanoma, *SSM* superficial spreading melanoma

^a^ Others = black, Asian or pacific islander, American indian/Alaska native

^b^ Other melanoma: International Classification of Diseases for Oncology-O-3, codes 8722–8723, 8730, 8740–8741, 8744–8746, 8761, 8770–8774, 8780

*P* values were obtained using the Pearson χ^2^ test or Fisher exact test. Bold values indicate statistical significance less than 0.05

### Identification of prognostic factors

**Fig. 4:**
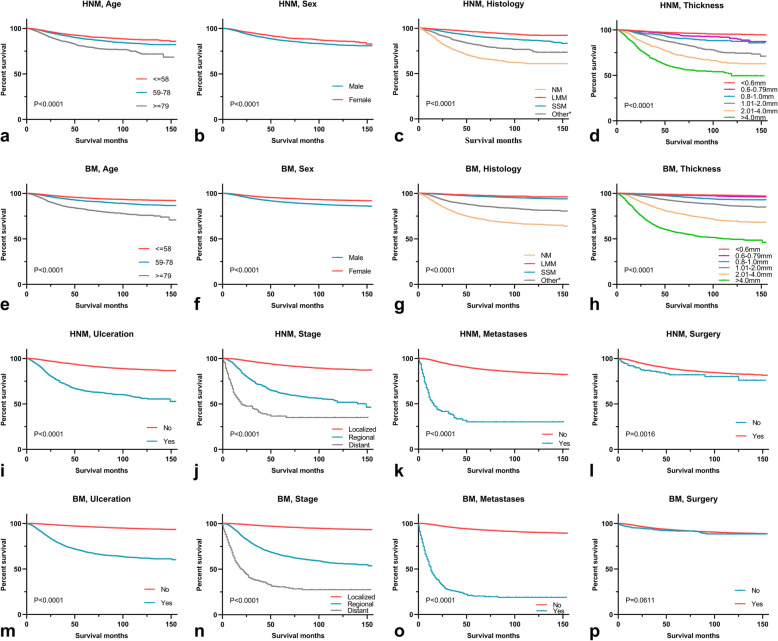
Kaplan-Meier curves of CSS were delineated based on the identified independent prognostic factors, including age, sex, histology, thickness, ulceration, stage, metastases, and surgery (**a**-**d**, **i**-**l**) for HNM training cohort, **e**-**h**, **m**-**p** for BM training cohort. CSS = cancer-specific survival; HNM = head and neck melanoma; BM = body melanoma

The univariate analysis revealed that nine variables (age, sex, race, histology, thickness, ulceration, stage, metastases, and surgery) were potential clinical determinants of CSS in the HNM group. In addition, the same nine significant factors were identified in the BM group. Table 3 shows the 5-year CSS and OS rates for the subgroups. The multivariable analysis revealed that the same eight variables (age, sex, histology, thickness, ulceration, stage, metastases, and surgery) were independent prognostic factors in both the HNM and BM training cohorts (Table 4). The survival curves for the identified independent factors of CSS for the HNM group and the BM group are illustrated in Fig. 4. The survival curves for OS in the two groups are shown in Figure S1.

**Table 3:** Univariate analysis of factors affecting cancer-specific and overall survival

Factors	5-year Cancer-Specific Survival rate				5-year Overall Survival rate			
	(%, mean ± SD)				(%, mean ± SD)			
	HNM	P value	BM	***P*** value	HNM	***P*** value	BM	***P*** value
**Age (CSS/OS)**		**< 0.001**		**< 0.001**		**< 0.001**		**< 0.001**
≤ 58 / ≤63	91.5 ± 0.5		94.8 ± 0.2		88.5 ± 0.5		92.7 ± 0.2	
59–78 / 64–79	88.3 ± 0.5		91.6 ± 0.3		75.7 ± 0.8		81.7 ± 0.4	
≥ 79 / ≥80	80.2 ± 1.1		82.6 ± 0.7		41.0 ± 1.2		48.8 ± 1.0	
**Sex**		**< 0.001**		**< 0.001**		**< 0.001**		**< 0.001**
Male	86.9 ± 0.4		90.5 ± 0.2		73.3 ± 0.6		82.7 ± 0.3	
Female	90.6 ± 0.6		94.8 ± 0.2		77.2 ± 0.9		89.7 ± 0.2	
**Race**		**0.011**		**< 0.001**		0.366		**< 0.001**
White	87.9 ± 0.4		92.8 ± 0.1		77.4 ± 0.5		86.2 ± 0.2	
^a^Others	82.3 ± 4.7		80.2 ± 1.8		71.5 ± 5.4		73.5 ± 2.0	
**Histology**		**< 0.001**		**< 0.001**		**< 0.001**		**< 0.001**
NM	67.7 ± 1.4		72.4 ± 0.7		53.7 ± 1.4		62.1 ± 0.7	
LMM	96.1 ± 0.4		97.7 ± 0.3		80.2 ± 0.8		86.2 ± 0.8	
SMM	91.0 ± 0.5		96.8 ± 0.1		79.9 ± 0.7		91.9 ± 0.2	
^b^Other Melanoma	81.9 ± 1.3		86.7 ± 0.6		68.3 ± 1.5		78.6 ± 0.7	
**Thickness**		**< 0.001**		**< 0.001**		**< 0.001**		**< 0.001**
< 0.6 mm	96.8 ± 0.3		98.6 ± 0.1		84.1 ± 0.6		93.3 ± 0.2	
0.6–0.79 mm	95.1 ± 0.8		98.1 ± 0.2		80.9 ± 1.4		92.8 ± 0.4	
0.8–1.0 mm	91.0 ± 1.2		96.6 ± 0.3		78.1 ± 1.6		91.4 ± 0.5	
1.01–2.0 mm	85.9 ± 1.0		91.6 ± 0.4		72.8 ± 1.2		85.6 ± 0.5	
2.01–4.0 mm	73.2 ± 1.5		78.5 ± 0.8		58.8 ± 1.6		68.2 ± 0.9	
> 4.0 mm	58.6 ± 1.8		57.5 ± 1.1		45.1 ± 1.7		46.0 ± 1.1	
**Ulceration**		**< 0.001**		**< 0.001**		**< 0.001**		**< 0.001**
No	92.2 ± 0.3		96.5 ± 0.1		79.1 ± 0.5		90.9 ± 0.2	
Yes	64.3 ± 1.4		69.1 ± 0.7		50.3 ± 1.4		58.5 ± 0.7	
**Stage**		**< 0.001**		**< 0.001**		**< 0.001**		**< 0.001**
Localized	92.7 ± 0.3		96.4 ± 0.1		78.7 ± 0.5		90.0 ± 0.2	
Regional	61.7 ± 1.6		65.5 ± 0.9		51.0 ± 1.6		58.2 ± 0.9	
Distant	36.6 ± 4.2		30.5 ± 2.4		28.4 ± 3.8		26.0 ± 2.3	
**Metastases**		**< 0.001**		**< 0.001**		**< 0.001**		**< 0.001**
No	88.7 ± 0.4		93.2 ± 0.1		75.1 ± 0.5		86.7 ± 0.2	
Yes	30.1 ± 4.5		20.3 ± 2.4		23.8 ± 4.2		17.6 ± 2.2	
**Surgery**		**0.002**		0.061		**< 0.001**		**< 0.001**
No	82.0 ± 3.0		91.6 ± 1.2		60.8 ± 3.8		80.7 ± 1.7	
Yes	88.0 ± 0.4		92.6 ± 0.2		74.7 ± 0.5		86.1 ± 0.2	

*HNM* head and neck melanoma, *BM* body melanoma, *SD* standard deviation, *CSS* cancer-specific survival, *OS* overall survival, *NM* nodular melanoma, *LMM* Lentigo maligna melanoma, *SSM* superficial spreading melanoma

^a^ Others = black, Asian or pacific islander, American indian/Alaska native

^b^ Other melanoma: International Classification of Diseases for Oncology-O-3, codes 8722–8723, 8730, 8740–8741, 8744–8746, 8761, 8770–8774, 8780

*P* values were obtained using the log-rank test. Bold values indicate statistical significance less than 0.05

**Table 4:** Multivariate analysis of factors affecting cancer-specific and overall survival

Factors	Cancer specific survival				Overall survival			
	HR (95% CI)				HR (95% CI)			
	HNM	***P*** value	BM	***P*** value	HNM	***P*** value	BM	***P*** value
**Age**								
≤ 58 / ≤63	Reference		Reference		Reference		Reference	
59–78 / 64–79	1.398 (1.211–1.613)	**< 0.001**	1.401 (1.289–1.522)	**< 0.001**	2.570 (2.318–2.849)	**< 0.001**	2.743 (2.583–2.912)	**< 0.001**
≥ 79 / ≥80	2.218 (1.891–2.602)	**< 0.001**	2.488 (2.237–2.766)	**< 0.001**	7.667 (6.918–8.497)	**< 0.001**	8.031 (7.519–8.578)	**< 0.001**
**Sex**								
Male	Reference		Reference		Reference		Reference	
Female	0.790 (0.686–0.908)	**0.001**	0.693 (0.641–0.749)	**< 0.001**	0.854 (0.784–0.930)	**< 0.001**	0.662 (0.629–0.698)	**< 0.001**
**Histology**								
NM	Reference		Reference		Reference		Reference	
LMM	0.538 (0.432–0.670)	**< 0.001**	0.566 (0.427–0.749)	**< 0.001**	0.795 (0.701–0.902)	**< 0.001**	0.988 (0.879–1.110)	0.841
SMM	0.834 (0.712–0.978)	**0.025**	0.615 (0.556–0.680)	**< 0.001**	0.851 (0.760–0.953)	**0.005**	0.724 (0.674–0.778)	**< 0.001**
^a^Other	0.607 (0.513–0.718)	**< 0.001**	0.813 (0.732–0.903)	**< 0.001**	0.655 (0.580–0.740)	**< 0.001**	0.811 (0.749–0.878)	**< 0.001**
**Thickness**								
< 0.6 mm	Reference		Reference		Reference		Reference	
0.6–0.79 mm	1.564 (1.141–2.144)	**0.005**	1.383 (1.102–1.735)	**0.005**	1.205 (1.037–1.401)	**0.015**	1.096 (0.987-1.217)	0.086
0.8–1.0 mm	2.156 (1.610–2.888)	**< 0.001**	2.255 (1.851–2.746)	**< 0.001**	1.264 (1.079–1.480)	**0.005**	1.235 (1.109–1.375)	**<0.001**
1.01–2.0 mm	3.292 (2.649–4.092)	**< 0.001**	3.192 (2.736–3.723)	**< 0.001**	1.558 (1.384–1.754)	**< 0.001**	1.446 (1.330–1.572)	**< 0.001**
2.01–4.0 mm	3.952 (3.146–4.965)	**< 0.001**	4.376 (3.724–5.142)	**< 0.001**	1.768 (1.552–2.015)	**< 0.001**	1.958 (1.785–2.147)	**< 0.001**
> 4.0 mm	4.417 (3.438–5.673)	**< 0.001**	5.613 (4.738–6.650)	**< 0.001**	2.080 (1.788–2.419)	**< 0.001**	2.524 (2.280–2.793)	**< 0.001**
**Ulceration**								
No	Reference		Reference		Reference		Reference	
Yes	1.842 (1.615–2.102)	**< 0.001**	2.225 (2.036–2.431)	**< 0.001**	1.472 (1.340–1.618)	**< 0.001**	1.775 (1.665–1.892)	**< 0.001**
**Stage**								
Localized	Reference		Reference		Reference		Reference	
Regional	2.547 (2.196–2.954)	**< 0.001**	3.179 (2.902–3.482)	**< 0.001**	1.677 (1.502–1.874)	**< 0.001**	2.044 (1.909–2.190)	**< 0.001**
Distant	3.683 (2.013–6.737)	**< 0.001**	5.245 (4.064–6.768)	**< 0.001**	2.434 (1.523–3.891)	**< 0.001**	3.667 (2.932–4.587)	**< 0.001**
**Metastases**								
No	Reference		Reference		Reference		Reference	
Yes	2.323 (1.237–4.364)	**0.009**	2.545 (1.939–3.342)	**< 0.001**	2.061 (1.244–3.413)	**0.005**	2.276 (1.779–2.912)	**< 0.001**
**Surgery**								
No	Reference		Reference		Reference		Reference	
Yes	0.369 (0.262–0.519)	**< 0.001**	0.536 (0.410–0.701)	**< 0.001**	0.568 (0.457–0.708)	**< 0.001**	0.510 (0.432–0.603)	**< 0.001**

*HNM* head and neck melanoma, *BM* body melanoma, *NM* nodular melanoma, *LMM* Lentigo maligna melanoma, *SSM* superficial spreading melanoma, *HR* hazard ratio, *95% CI* 95% confidence interval, *CSS* cancer-specific survival, *OS* overall survival

^a^ Other: International Classification of Diseases for Oncology-O-3, codes 8722–8723, 8730, 8740–8741, 8744–8746, 8761, 8770–8774, 8780

Bold values indicate statistical significance less than 0.05

### Nomogram development

**Fig. 5:**
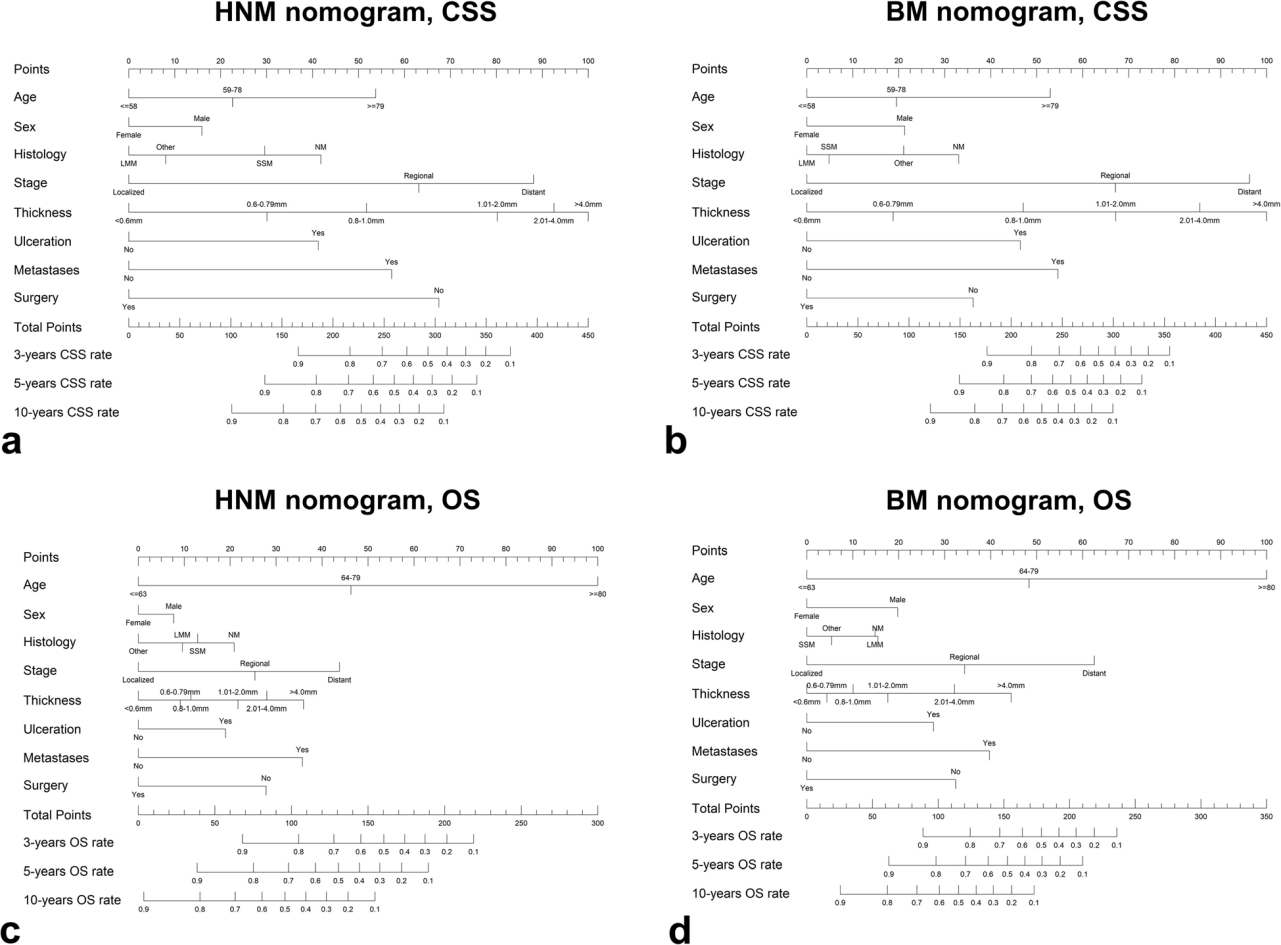
Nomograms predicted the individual 3-, 5- and 10-year survival rates of patients. **a**, **c** Predicting CSS and OS for HNM patients. **b**, **d** Predicting CSS and OS for BM patients. CSS = cancer-specific survival; OS = overall survival; HNM = head and neck melanoma; BM = body melanoma

The eight independent prognostic factors identified in the multivariate survival analyses were used to construct nomograms for HNM patients or BM patients to predict 3-, 5-, and 10-year CSS and OS (Fig. 5).

The nomogram of HNM showed that thickness (> 4.0 mm), which had the largest absolute values, was the strongest contributor to the risk of CSS prognosis, followed by distant stage, no surgery, having metastases and age > 79 years. Notably, in the BM nomogram, thickness (> 4.0 mm) contributed to the highest CSS risk, but distant stage contributed similarly to thickness. Present metastases, age over 79 years and ulceration were strong factors after distant stage in the BM nomogram of CSS.

By adding the scores of each predictor based on individual conditions (Table 5), the HNM nomogram and BM nomogram can be used to predict the 3-, 5- and 10-year CSS and OS rates for HNM patients and BM patients, respectively. For example, Patient A is a 65-year-old male patient who had a head (scalp) tumor thickness of 3.0 mm and was diagnosed with localized SSM without ulceration and metastases and then underwent surgery. A total of 160.6 points were given to this patient, and the estimated 10-year CSS rate was 77%. Patient B, who had all the same clinical features as patient A and underwent surgery but had a tumor site on the body (shoulder), had a total of 131.08 points. This patient’s predicted 10-year CSS rate was 88%, which was 11% higher than the CSS rate of patient A.

**Table 5:** Detailed scores of predictors in nomograms

Factors	CSS nomogram		OS nomogram	
	HNM	BM	HNM	BM
**Age (CSS/OS)**				
≤ 58 / ≤63	0	0	0	0
59–78 / 64–79	22.59	19.51	46.27	48.33
≥ 79 / ≥80	53.72	52.93	100.00	100.00
**Sex**				
Female	0	0	0	0
Male	15.92	21.29	7.73	19.75
**Histology**				
NM	41.82	33.05	20.87	15.46
LMM	0	0	9.59	14.85
SSM	29.56	4.83	12.91	0
^a^Other Melanoma	8.04	21.05	0	5.40
**Thickness**				
< 0.6 mm	0	0	0	0
0.6–0.79 mm	30.07	18.77	9.12	4.37
0.8–1.0 mm	51.72	47.07	11.45	10.07
1.01–2.0 mm	80.19	67.17	21.69	17.62
2.01–4.0 mm	92.53	85.45	27.95	32.14
> 4.0 mm	100.00	100.00	35.95	44.43
**Ulceration**				
No	0	0	0	0
Yes	41.25	46.43	19.01	27.56
**Stage**				
Localized	0	0	0	0
Regional	63.1	67.12	25.39	34.32
Distant	88.05	96.29	43.79	62.52
**Metastases**				
No	0	0	0	0
Yes	57.21	54.61	35.73	39.7
**Surgery**				
Yes	0	0	0	0
No	67.49	36.18	27.83	32.35

*HNM* head and neck melanoma, *BM* body melanoma, *CSS* cancer-specific survival, *OS* overall survival, *NM* nodular melanoma, *LMM* Lentigo maligna melanoma, *SSM* superficial spreading melanoma

^a^ Other melanoma: International Classification of Diseases for Oncology-O-3, codes 8722–8723, 8730, 8740–8741, 8744–8746, 8761, 8770–8774, 8780

In addition, to optimize the calculation process, we built an operation interface of every nomogram on the web page [33–36]. By entering a patient’s condition and choosing a survival time to be predicted, the user can obtain predictive results.

### Validation and calibration of the nomogram

**Fig. 6:**
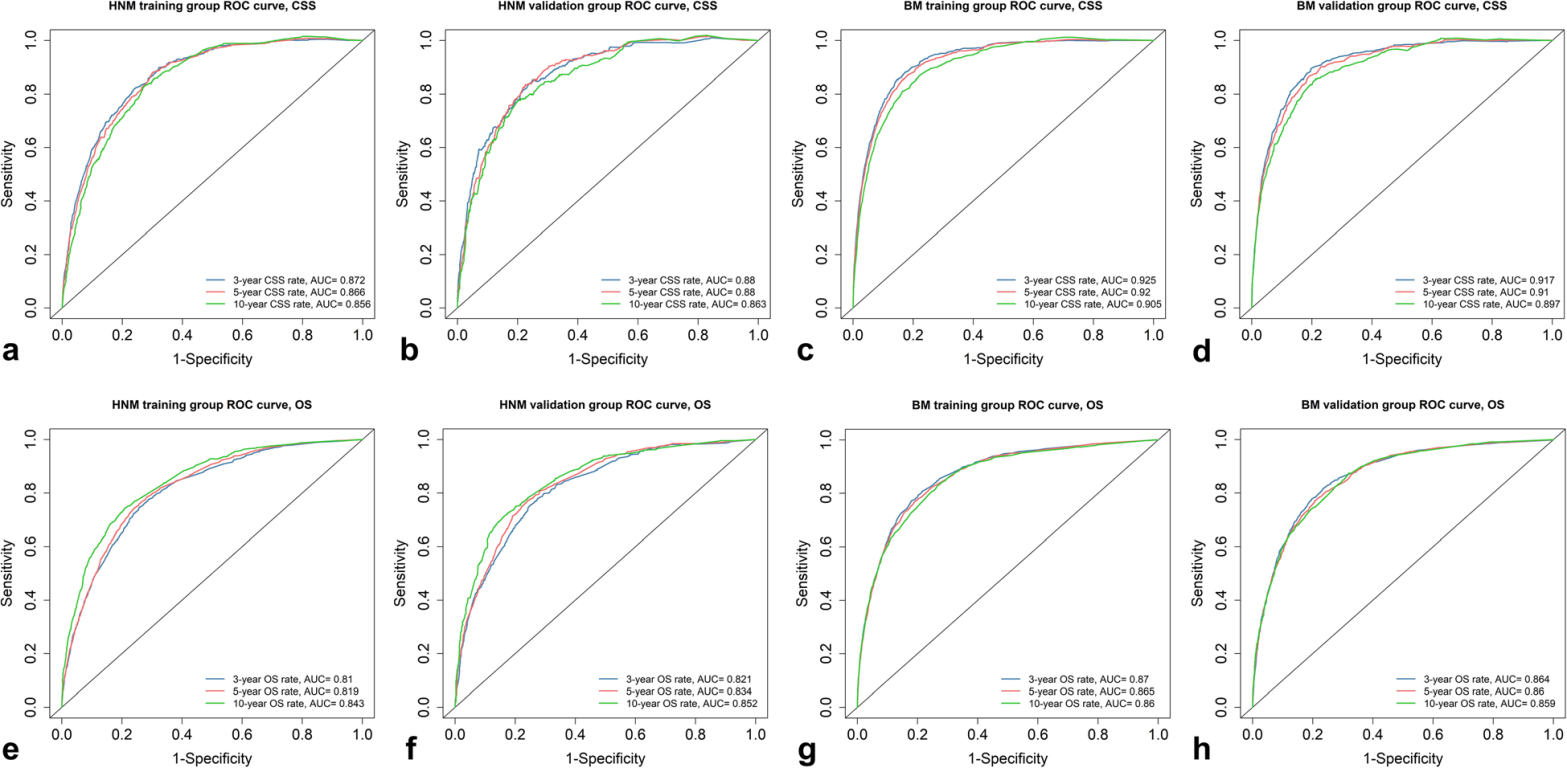
ROC curves evaluated the predictive ability of the nomograms. **a**-**d** Predicting 3-, 5- and 10-year CSS rates for HNM training and validation cohort, BM training and validation cohort, respectively. **e**-**h** Predicting 3-, 5- and 10-year OS rates for HNM training and validation cohort, BM training and validation cohort, respectively. CSS = cancer-specific survival; OS = overall survival

**Fig. 7:**
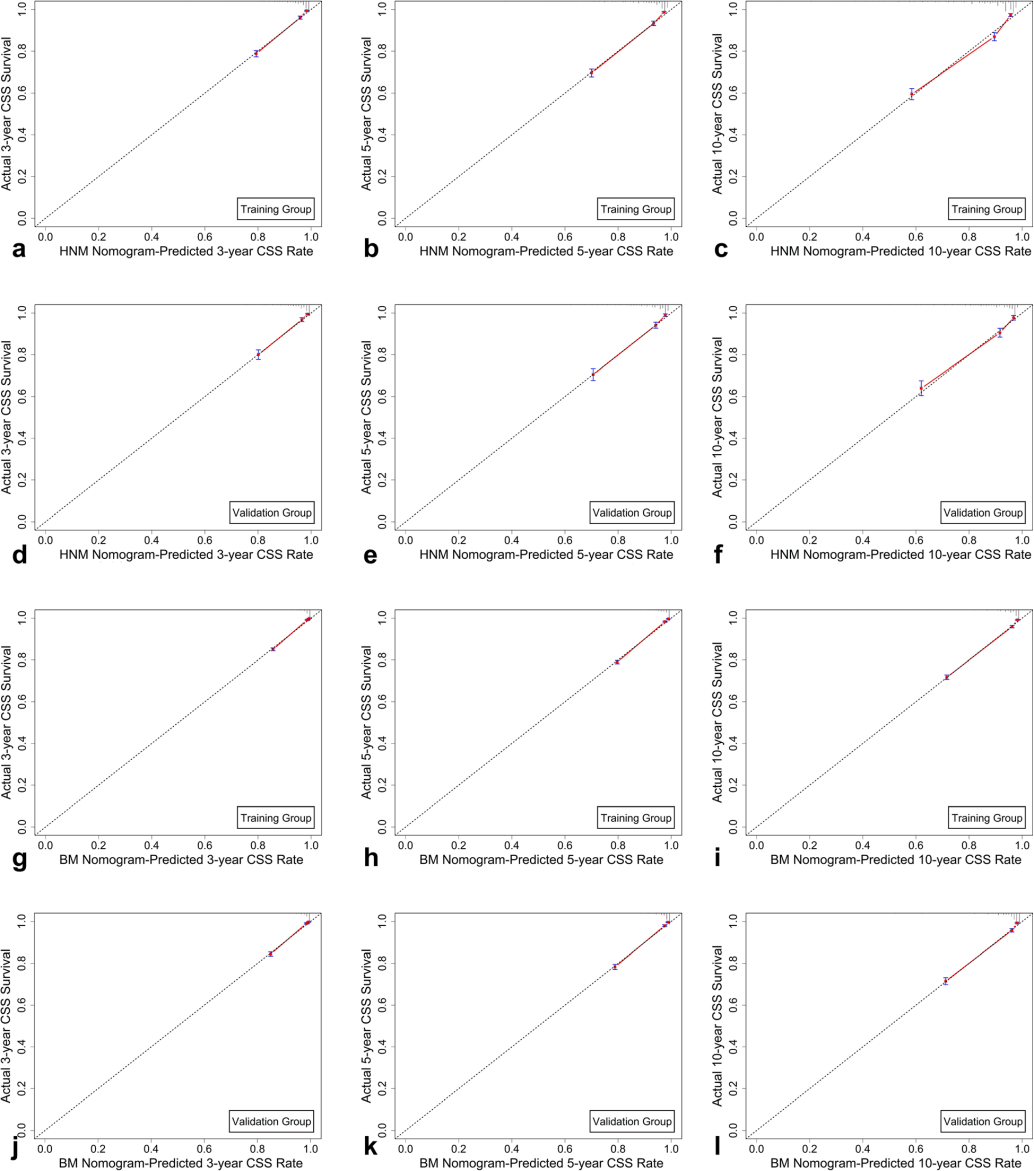
Calibration curves showed the probability of 3-, 5- and 10-year cancer-specific survival (CSS) between the nomogram prediction and the actual observation. Perfect prediction would correspond to the 45-degree line. The calibration curves predicted CSS of patients with HNM in the training cohort (**a**-**c**) and in the validation cohort (**d**-**f**). The calibration curves predicted CSS of patients with BM in the training cohort (**g**-**h**) and in the validation cohort (**j**-**l**)

The predictive ability of each nomogram was validated both internally (training cohort) and externally (validation cohort) by the C-index and AUC values. The C-indexes of the four nomograms ranged from 0.785 to 0.896 in the internal validation cohort and from 0.796 to 0.889 in the external validation cohort, showing good accuracy and discrimination of the models (Table 6). The AUCs for the 3-, 5- and 10-year CSS and OS rates in each training and validation cohort were all high, ranging from 0.81 to 0.925 (Fig. 6). In addition, the calibration plots for the probability of 3-, 5- and 10-year CSS revealed great consistency between the nomogram-based prediction and the actual observed outcomes (Fig. 7). Similar results were found in the calibration of the OS prediction nomograms (Figure S2). These results indicate that these four nomograms exhibit excellent performance for predicting the survival of malignant cutaneous melanoma patients.

**Table 6:** The concordance index of nomograms for internal and external validation

	HNM nomogram validation		BM nomogram validation	
	Internal	External	Internal	External
**CSS**	0.839 ± 0.005	0.848 ± 0.008	0.896 ± 0.003	0.889 ± 0.004
**OS**	0.785 ± 0.004	0.796 ± 0.006	0.842 ± 0.002	0.840 ± 0.004

*HNM* head and neck melanoma, *BM* body melanoma, *CSS* cancer-specific survival, *OS* overall survival

## Discussion

In this study, we compared the characteristics of HNM patients with those of BM patients based on the data of a large SEER population. Moreover, we were the first to construct and validate nomograms for predicting the CSS and OS rates of patients with HNM or BM. We reported a relatively high incidence of HNM, suggesting the importance of a tumor located in the head and neck to public health. Ten factors were significantly different between HNM and BM, suggesting that HNM and BM are heterogeneous. Dabouz et al. also reported that HNM differed from BM by age, histology and thickness [9]. The survival rate of HNM patients was notably lower than that of BM patients in this study, and we recommend that cutaneous melanomas in the head and neck region be paid close attention and be managed carefully.

Each nomogram developed in our study included two personal variables (age and sex), five melanoma-related variables (histology, thickness, ulceration, stage and metastases), and one therapy-related variable (surgery). Previous studies reported that advanced age and male sex were associated with a poor prognosis of melanoma, and HNM occurred more frequently in elderly individuals and male patients, consistent with our research [37–39]. Older patients are likely to be in poorer physical conditions and have chronic diseases; thus, tumor burden cannot be tolerated. Elderly individuals usually suffer longer chronic sun exposure than younger individuals in regions of the head and neck, may explaining the higher HNM rate. In melanoma, the female survival benefit may result from the protective role of estrogen [40, 41]. Usually, women are more active in the prevention of sun exposure on the head and neck, which may reduce the risk of HNM [8]. Histology has been reported as an important survival factor for melanoma patients [42, 43]. In agreement with other studies, we observed a high frequency of LMM on the head and neck [7, 9]. This may due to the different patterns of sun exposure between the head/neck and other anatomical locations [44, 45]. Thickness is also an independent prognostic factor for melanoma, and increasing thickness implies a worse prognosis [31]. In our study and in other studies, thicker tumors were more common on the head and neck than other areas [14, 15]. This may be partly explained by the late diagnosis, as HNM may be hidden by hair. In addition, we found that the 5-year survival rate of HNM patients with a tumor diameter of 0.6–0.79 mm was lower than that of patients with a tumor diameter < 0.6 mm, but similar results were not obtained in BM patients, which may suggest that the risk of death is increased when the thickness of the HNM is larger than 0.6 mm. These findings show that patients with HNM require close follow-up. Ulceration, lymph node stage and metastasis have been identified as significant independent factors of cutaneous melanoma in previous studies and were also identified in our study [26, 28]. The capillary vessels and lymphatic drainage systems on the head/neck are rich and complex, and these characteristics may facilitate the growth of ulcerations, distant stages and tumor metastases [27, 46]. In addition, HNM is prone to ulcers and metastasis may result as it tends to be diagnosed in the late stage and lacks early treatment. Surgery is the main therapy for melanoma and can improve survival [47]. However, even after receiving surgical treatment, the survival rates in patients with HNM were lower than those with BM, which may be because HNM patients tend to be older and fail to tolerate surgery or postoperative complications [48]. Another reason is that HNM may not receive an adequate margin resection for cosmetic or functional reasons [49].

These nomograms can be easily used for to estimate survival in individuals, to counsel patients and to guide therapy. By calculating the sum of the scores, clinicians can use the nomogram to provide a quantified survival prediction for individual HNM or BM patients. Notably, with a simple operation interface, the nomograms on the web page can offer an accurate and quick personal prognosis, improving clinical practicability. In addition, both the C-index and AUC ranged from 0.5 (no discrimination) to 1 (perfect discrimination). The internal and external validation of each nomogram in this study showed high C-index values (0.785–0.896) and AUC values (0.81–0.925), indicating that our models have great predictive and discriminatory power. The calibration curves suggested satisfactory consistency of the nomogram. In general, our nomograms can be used as handy clinical tools to estimate the CSS and OS of patients with HNM or BM.

This study has the following limitations that need to be acknowledged. First, we grouped melanoma on the head and neck together as HNM for analysis and did not investigate specific anatomic subsites in this region individually. A similar problem existed in the BM analysis. Previous studies suggested that some precise areas had worse cutaneous melanoma-specific prognoses, such as the scalp and breast [3, 5]. Second, our patients were limited to the SEER program, and the results of our study might not be generalizable to other regional groups. Future studies are needed to validate the proposed nomograms. Third, we could not obtain precise data on radiotherapy and chemotherapy from the SEER database, as patients only had “Yes” or “None/Unknown” labels for these variables; thus, only surgery was included in the survival analysis. In addition, some variables like BRAF status and lactate dehydrogenase (LDH) level were unavailable or deficient in SEER program, which have been reported as important prognostic factors [31, 47]. Finally, as a retrospective study, it is susceptible to some inherent biases.

## Conclusion

The characteristics of HNM and BM are heterogeneous, and patients with HNM had a worse prognosis in this study. We constructed and validated four nomograms for predicting the 3-, 5- and 10-year CSS and OS probabilities of patients with HNM or BM. These nomograms, with accurate predictive power, can serve as useful and convenient clinical tools that facilitate prognosis prediction, personalized melanoma consultation and individual health management.

## Supplementary Information

**Additional file 1: Figure S1.** Kaplan-Meier curves of OS. **Figure S2.** Calibration curves for nomograms of OS.

## Data Availability

The data were collected from the Surveillance, Epidemiology, and End Results (SEER) database (URL: https://seer.cancer.gov/, Accession username:12814-Nov2019).

## Abbreviations

HNM: Head and neck melanoma
BM: Body melanoma
SEER: Surveillance, Epidemiology, and End Results
OS: Overall survival
CSS: Cancer-specific survival
AJCC: American Joint Committee on Cancer
HR: Hazard ratios
CIs: Confidence intervals
C-index: The concordance index
AUC: The area under the curve
ROC: The receiver operating characteristic
SSM: Superficial spreading melanoma
LMM: Lentigo maligna melanoma
LDH: Lactate dehydrogenase
