# Supplementary material for: Comparing the characteristics and predicting the survival of patients with head and neck melanoma versus body melanoma: a population-based study
Source: BMC Cancer. 2021 Apr 16;21:420. doi: 10.1186/s12885-021-08105-y (PMC8052690; doi:10.1186/s12885-021-08105-y)

***Supplementary Material***

***Title Page:***

**Comparing the Characteristics and Predicting the Survival of Patients with Head and Neck Melanoma versus Body Melanoma: A Population-based Study**

Yuxin Ding, MD^1#^, Runyi Jiang, MD^2#^, Yuhong Chen, MD^1^, Jing Jing, PhD^1^, Xiaoshuang Yang, MD^1^, Xianjie Wu, PhD^1^, Xiaoyang Zhang, MD^1^, Jiali Xu, MD^1^, Piaopiao Xu, MD^1^, ShuChen LiuHuang, MFA^3^, Zhongfa Lu, PhD, MD^1*^

^1^ Department of Dermatology, The Second Affiliated Hospital, School of Medicine, Zhejiang University, Hangzhou, China, No. 88, Jiefang Road, Hangzhou 310009, China

^2^ Spinal Tumor Center, Department of Orthopaedic Oncology, Changzheng Hospital, Second Military Medical University, Shanghai, 200003, China

^3^ China Academy of Art, Hangzhou, 310000, China

# Yuxin Ding, Runyi Jiang Contributed equally to this work.

***Corresponding author:**

Zhongfa Lu, Department of Dermatology, The Second Affiliated Hospital, School of Medicine, Zhejiang University, Hangzhou, China, No. 88, Jiefang Road, Hangzhou 310009, China.

TEL: +86-137-5712-8317; E-mail: lzfskin@zju.edu.cn

***Supplementary Figure***

**Fig. S1** Kaplan-Meier curves of OS were delineated based on the identified independent prognostic factors, including age, sex, histology, thickness, ulceration, stage, metastases, and surgery **(a-d, i-l)** for HNM training cohort, **(e-h, m-p)** for BM training cohort.

OS = overall survival; HNM = head and neck melanoma; BM = body melanoma


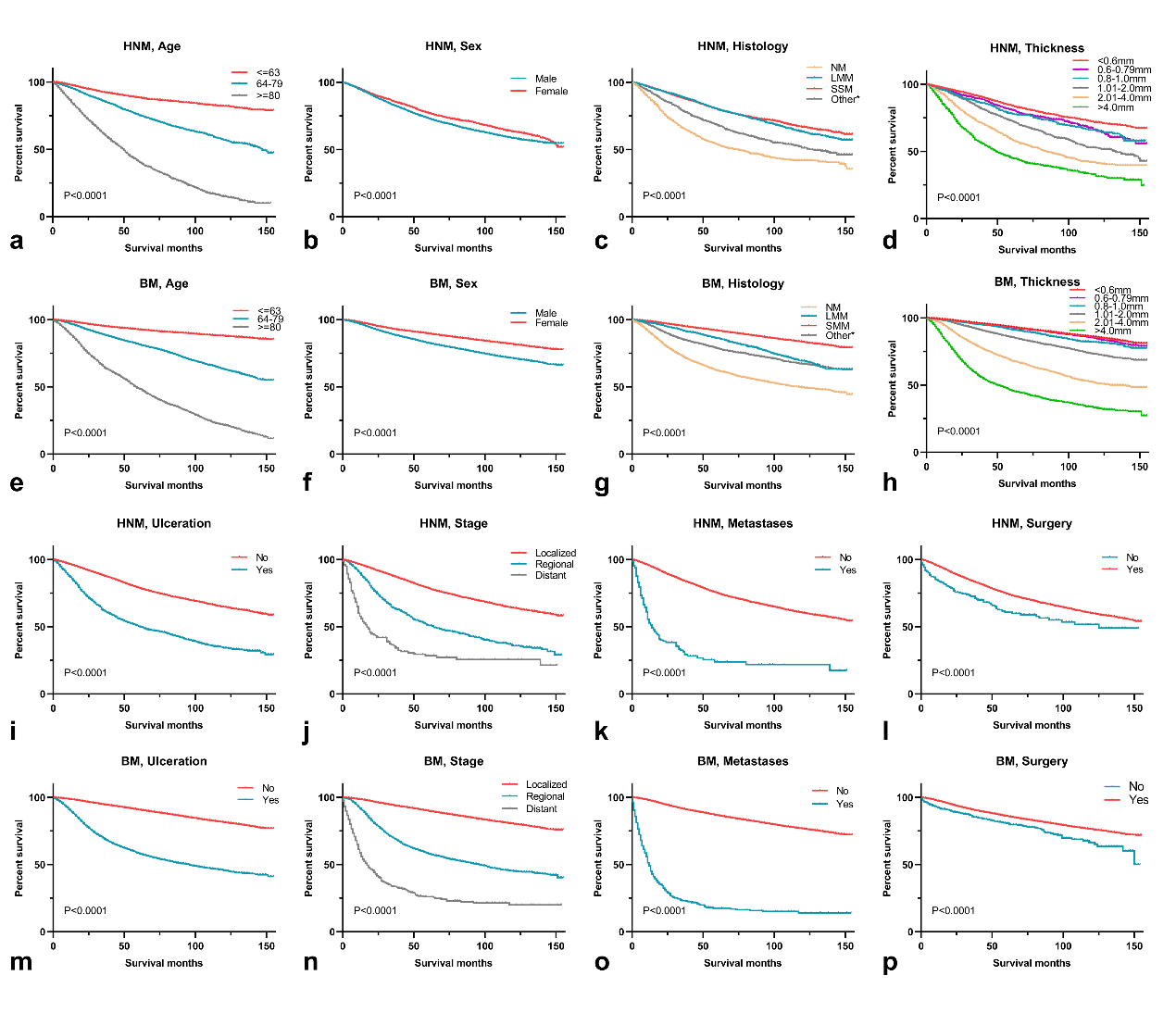


**Fig. S2** Calibration curves showed the probability of 3-, 5- and 10-year overall survival (OS) between the nomogram prediction and the actual observation. Perfect prediction would correspond to the 45-degree line.

The calibration curves predicted OS of patients with HNM in the training group **(a-c)** and in the validation group **(d-f).** The calibration curves predicted OS of patients with BM in the training group **(g-h)** and in the validation group **(j-l)**.


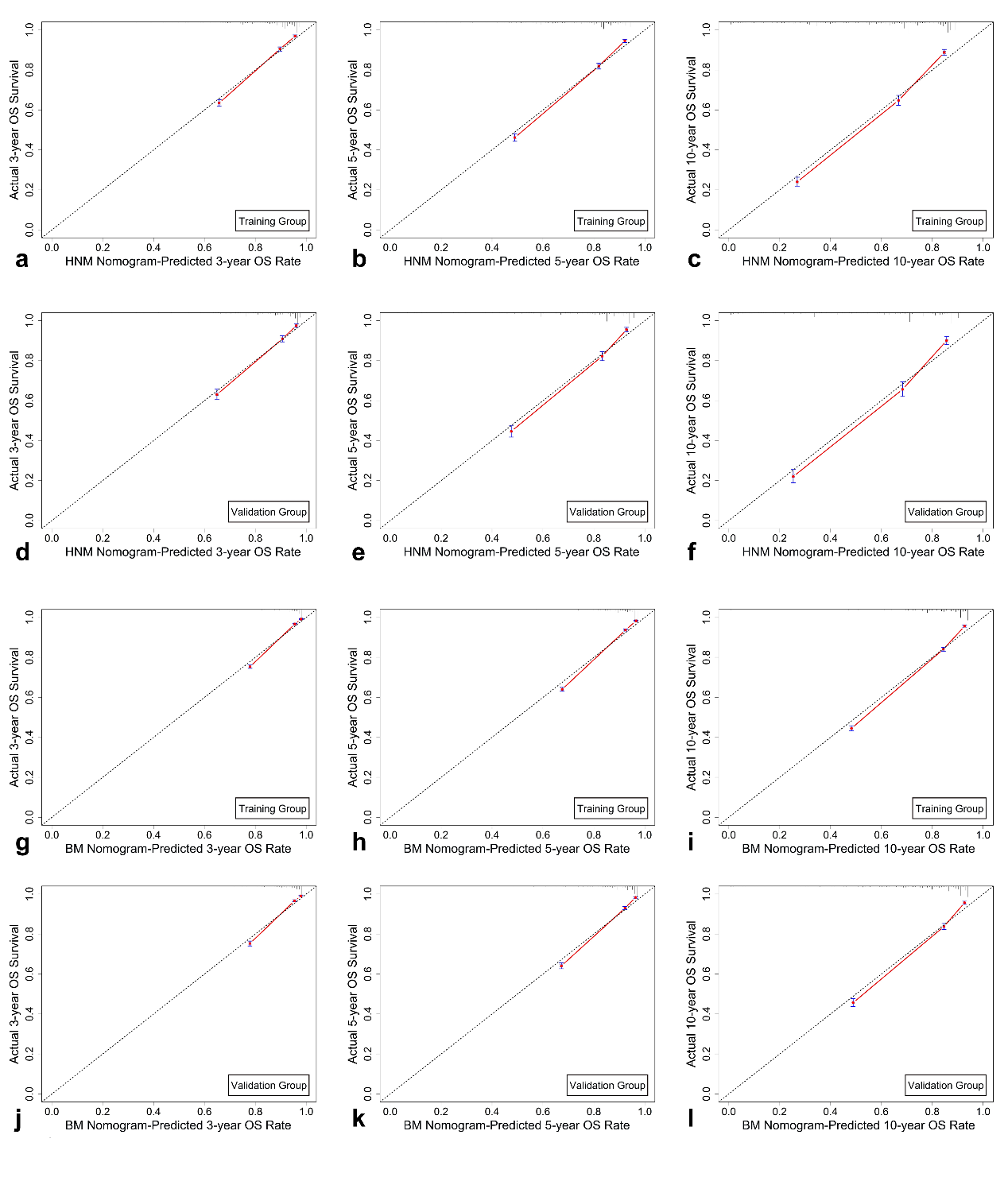

Supplement: Supplementary file 1 — Additional file 1: Figure S1. Kaplan-Meier curves of OS. Figure S2. Calibration curves for nomograms of OS. [file 12885_2021_8105_MOESM1_ESM.docx]
